# Patterns of tobacco and e-cigarette use status in India: a cross-sectional survey of 3000 vapers in eight Indian cities

**DOI:** 10.1186/s12954-020-00362-7

**Published:** 2020-03-30

**Authors:** Rajeshwar Nath Sharan, Tongbram Malemnganbi Chanu, Tapan Kumar Chakrabarty, Konstantinos Farsalinos

**Affiliations:** 1 Radiation & Molecular Biology Unit, Department of Biochemistry, North-Eastern Hill University, Shillong, 793022 India; 2 Department of Statistics, North-Eastern Hill University, Shillong, 793022 India; 3 Department of Cardiology, Onassis Cardiac Surgery Center, Sygrou 356, 17674 Kallithea, Greece; 4 Center of Excellence in Trauma and Accidents, King Abdulaziz University, Jeddah, Saudi Arabia; 5 Department of Pharmacy, University of Patras, 26500 Rio, Greece

**Keywords:** Tobacco smoking, E-cigarette, Nicotine, Vaping, Tobacco cessation

## Abstract

**Background:**

Tobacco smoking is one of the biggest and avoidable public health threats in the world, especially in low- and middle-income countries. India represents a highly complex public health environment due to the large number of smokers and complexities arising from tobacco use in different forms, including a variety of smokeless tobacco (SLT) products. Electronic cigarettes, an alternative nicotine delivery system with significantly less harmful emissions than smoke, could be an option for those who are unable to achieve smoking abstinence using other available means. This study, which we believe is the first of this kind in India, was conducted to obtain data on the characteristics and tobacco use profile of e-cigarette users (vapers) in India.

**Methods:**

An interview-based survey was performed in the 8 largest metropolitan cities in India using a convenience sampling approach involving a total of 3000 subjects. Inclusion criteria were being a current e-cigarette user and aged 18 years or more. Interviewers were asked to continue recruitment until a total sample of 375 was reached in each target city.

**Results:**

A total of 3000 vapers (81.4% males and 18.6% females, median age 29 years) participated to the study. The majority (80%) were first exposed to nicotine via tobacco smoking, SLT use, or both. Most of the subjects (79%) believed that e-cigarettes were less harmful than smoking. The vast majority of smokers (71.3%) reported smoking cessation (30.0%) or reduction in consumption (41.3%) with the help of e-cigarettes. Similar changes were observed in SLT users. Participants reported minimal side effects and some health benefits after e-cigarette use initiation.

**Conclusion:**

Indian vapers who participated to this study were predominantly smokers and SLT users before e-cigarette use initiation, with the majority subsequently quitting or reducing tobacco use. Minimal side effects of e-cigarette were experienced, while some health benefits were also reported.

## Introduction

Tobacco smoking is one of the biggest preventable causes of non-communicable disease affecting both users and bystanders [1, 2]. The World Health Organization (WHO) estimates that there are more than 1.1 billion smokers globally and predicts 1 billion premature smoking-related deaths during the twenty-first century. Approximately, 80% of these deaths are likely to occur in the low- and middle-income countries (LMICs) where the prevalence of smoking is particularly high and growing [2]. India, the world’s fastest growing large economy in 2018, is the home to over 11% of the world’s cigarette smokers but also has a significantly larger proportion of the population indulging in (a) smoking tobacco in its alternative or local forms (e.g., *bidis*, *hookah*, *chilam*, *shisha*, water pipes), (b) chewing or masticating smokeless tobacco (SLT) in various forms, e.g., *khaini*, *zarda*, *gutkha*, and *paan masala* in combination with or without betel (*Areca*) nut, or (c) a combination of the two (mixed users). Therefore, India represents a complex public health challenge [3]. Tobacco-related deaths in India are estimated to be over 1 million/year [4] and are projected to rise to 1.5 million by 2020 [5]. Global Adult Tobacco Survey (GATS) data of 2016–2017 shows that India has the second largest tobacco consuming population in the world, estimated to be over 267 million, which includes at least 100 million tobacco smokers and over 199 million SLT users [6]. While smoking tobacco in different forms, including cigarettes, is predominantly found among men, SLT usage is more widespread among women [6].

The impact of smoking and harmful SLT use in India is evident from data on disease burden in the Indian population [7]. While ischemic heart disease and chronic pulmonary obstructive disease, diseases that are etiologically linked to smoking [8, 9] were ranked 6th and 8th, respectively; in disease burden in India during the 1990s, they were in the first two positions in 2016 [7]. Therefore, reducing the prevalence of smoking and other harmful tobacco product use is expected to have a substantial public health impact in India. However, the country faces substantial challenges related to the lack of adequate facilities to support smoking cessation [10], limited recruitment and success among smokers [11], and limited motivation and experience of healthcare professionals to provide smoking cessation support [12, 13].

A relatively recent technological development is the invention of electronic cigarettes (e-cigarettes), products that can deliver nicotine without combustion. These can be used as a harm reduction tool and appear to be a viable option for those who are unable to quit smoking using other available means [1, 14]. E-cigarettes have undergone significant refinements and improvements from the first generation devices of early 2005 to the currently available third and fourth generation improved devices offering better performance [15–19].

The entry of e-cigarettes to the Indian market triggered debate among users, clinicians, scientists, public health professionals, policy makers, and other stakeholders on the pros and cons of e-cigarette use as an additional tool to achieve better tobacco control. E-cigarettes are currently being used by only a very small proportion of Indians. The 2015 GATS-2 estimated that the prevalence of use was 0.02% (268,000 users) [6]. The Indian Council of Medical Research has expressed substantial concerns about e-cigarettes and recommended a complete prohibition of e-cigarettes to protect public health [20]. While there has been some criticism that the report was unbalanced and failed to consider the totality of the evidence [21], the Indian government decided to implement a total ban on the import and sale of these products. Unfortunately, these decisions were made without previously examining the patterns of e-cigarettes use in India and the profile, smoking status, and perceived benefits or harms among local users. To better understand such parameters, we performed the first, to the best of our knowledge, cross-sectional survey among e-cigarette users (vapers) in India.

## Methods

**Fig. 1:**
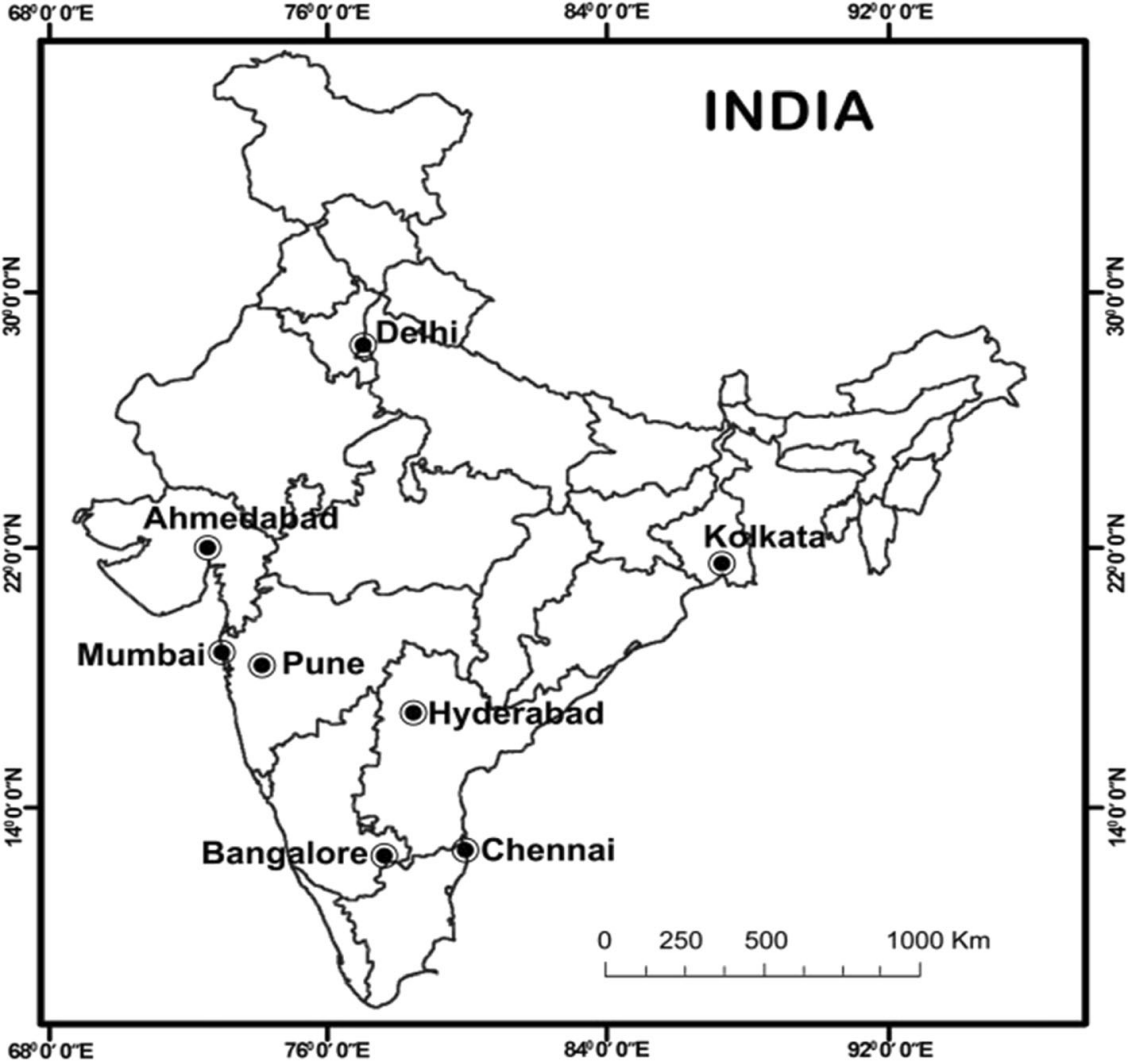
Map of India showing the geographical locations of the 8 target metropolitan cities covered in this study

Since e-cigarette use was low in India, it was not feasible to recruit vapers using a probability-based random sampling approach. There were also no suitable sampling frames in India (e.g., membership lists) from which to select subjects. Many surveys of vapers have relied on self-volunteered internet samples, but these also face various limitations (lack of control over recruitment location, possibility for double entries, confirmation of respondent’s fit into the inclusion criteria, indeterminate reliability and validity, etc.). In this study, we aimed to generate a sample of current vapers in India in order to obtain information on their experience with e-cigarette use and examine their past and current smoking status. Therefore, and in light of the limitations of sampling avenues, we decided to recruit a convenience sample of e-cigarette users from across India, in order to reduce bias due to recruitment in one location. We chose the 8 largest metropolitan areas of India, namely Delhi in northern, Kolkata in the eastern, Ahmedabad, Mumbai, and Pune in western, and Bangalore, Chennai, and Hyderabad in the southern regions of India (Fig. 1). We decided on an overall sample size of 3000 in order to increase the power to conduct analysis of subgroups (e.g., gender, prevalence, and health). This was a convenience sample in which interviewers in these target cities were asked to identify vapers from among their contacts and networks. Inclusion criteria were being a current e-cigarette user (based on self-report) and aged ≥ 18 years. Interviewers were asked to continue recruitment until a total sample of 375 was reached in each target city.

A questionnaire comprising 72 questions was used for the survey, which was based on previous online surveys [22, 23]. To improve the questionnaire design and ensure the comprehension of the survey items, 7 e-cigarette users (who did not participate to the final study sample) were recruited and participated in an assessment of the questionnaire using the method of cognitive interviewing [24]. The first part of the questionnaire included demographic data. Subsequently, participants were asked about their past (before e-cigarette use initiation) and current smoking and SLT use patterns, including duration and frequency of use. Assessment of smoking dependence was performed by using the Fagerström Test for Cigarette Dependence (FTCD) [25]. Then, there were questions about patterns of current e-cigarette use, including frequency of use, types of products used, and daily consumption. Additionally, participants were asked to report self-perceived changes in health parameters as well as side effects after e-cigarette use initiation, based on a previous survey [22]. The self-reported effect of e-cigarettes on tobacco use status of study participants was assessed separately for smoking tobacco and for SLT use by asking “Did you manage to reduce or quit smoking tobacco with the help of e-cigarettes?” and “Did you manage to reduce or quit chewing (oral) tobacco with the help of e-cigarette?” Response options were (1) Yes, I managed to quit; (2) Yes, I managed to reduce consumption; (3) Initially, I managed to quit but then I relapsed; (4) No, I did not quit or reduce consumption; and (5) No and in fact I increased my consumption. Finally, the perception of harm from e-cigarette was examined both in absolute terms and in comparison to smoking by asking “Do you think e-cigarettes are (1) Absolutely harmless, (2) Substantially less harmful than tobacco cigarettes, (3) Slightly less harmful than tobacco cigarettes, (4) Equally harmful as tobacco cigarettes, 5. Slightly more harmful than tobacco cigarettes, and (6) Substantially more harmful than tobacco cigarettes.”

The questionnaire was loaded on handheld devices, laptops, or PCs and was pre-tested before its application in the field. Interviews were conducted either face-to-face using the handheld devices or laptops, or in CATI (computer-assisted-telephone-interview) mode. Field-level data were aggregated and perused weekly for quality checks.

The study was carried out as per the guidelines approved by the Institutional Ethics Committee for Human Samples/Participants (IECHSP) of NEHU. The study was approved on August 02, 2017.

## Statistical analysis

Descriptive analysis was performed, with continuous variables reported as median (interquartile range [IQR]) and categorical variables reported as number (percentage). Non parametric tests (McNemar test) were used to compare smoking and SLT use prevalence between the time before e-cigarette use initiation and the time of the survey while smoking consumption was compared between these two time-points using Wilcoxon signed rank test. Binary logistic regression analysis was performed to examine correlates of having quit smoking. The dependent variable was being a former (rather than a current) smoker, and independent variables were age, gender, education level, smoking duration and consumption, FTCD, e-cigarette use duration, frequency of e-cigarette use, liquid consumption, nicotine concentration used, number of different flavors used (one vs. two or more), type of flavors used (tobacco/mint/menthol only vs. at least one non-tobacco/mint/menthol flavor), and perceptions of e-cigarette harmfulness (equally/more harmful than smoking vs. harmless/less harmful than smoking). All data were analyzed using the SPSS ver. 22.0 software.

## Results

### Participant characteristics and tobacco use before e-cigarette use initiation

A total of 3000 respondents were recruited in equal numbers (*n* = 375) from each of the 8 metropolitan cities. The demographics characteristics and history of tobacco use before e-cigarette use initiation of the study participants are presented in Table 1. The majority of the study sample (81.4%) was men. The median age of the respondents was 27 years for women and 30 years for men. Most of them were educated at graduate level or higher. Most participants (71.4%) had their first exposure to nicotine through combustible tobacco and 9.5% through SLT. Of the remaining 19.1% respondents, 1.6% reported having their first exposure to nicotine through pharmaceutical products and 17.5% through e-cigarettes. Most participants reported smoking tobacco before e-cigarette use initiation, and they had low to moderate cigarette dependence as assessed by FTCD. A small proportion of participants were SLT users before e-cigarette use initiation. However, the vast majority of these subjects were also smoking tobacco; only 1.5% of SLT users (*n* = 6) reported not smoking tobacco.

**Table 1:** Participant demographics and tobacco use before e-cigarette use initiation

	*n* (%) or median (IQR)
Participants	3000 (100)
Residence	
Eastern India	375 (12.5)
Western India	1125 (37.5)
Northern India	375 (12.5)
Southern India	1125 (37.5)
Age (years)	29 (25–35)
Gender	
Male	2443 (81.4)
Female	557 (18.6)
Education	19,269
Less than Senior School Certificate	6 (0.2)
Secondary/High School Certificate	340 (11.3)
Graduate/postgraduate	1874 (62.5)
Professional degree	780 (26.0)
First nicotine product ever tried	
Combustible tobacco^1^	2143 (71.4)
Chewing (oral) tobacco	284 (9.5)
Pharmaceutical nicotine product	49 (1.6)
E-cigarette	524 (17.5)
Smoking before e-cigarette use initiation	2350 (78.3)
Boxed or roll-your-own cigarettes	2002 (66.7)
Beedies	33 (1.1)
Both	315 (10.5)
Smoking duration (months)	72 (50–108)
Daily smoking consumption before e-cigarette use initiation	8 (6–10)
FTCD before e-cigarette use initiation	4 (3–5)
SLT use before e-cigarette use initiation	396 (13.2)
Duration of SLT use	60(37–93)
SLT consumption (times per day)	5 (4–7)
SLT users who also smoked tobacco	390 (98.5)

^1^The question defined combustible tobacco as “Cigarettes, Cigarillos, Beedies, Hukka/Pipe/Shisha, Cigar, etc.”

*FTCD* Fagerstrom Test for Cigarette Dependence, *SLT* smokeless tobacco

### Current tobacco use patterns

Current tobacco use patterns of study participants are presented in Table 2. More than half of all participants were still smoking tobacco, but smoking prevalence was reduced from 78.3% before e-cigarette use initiation to 58.4% at the time of the survey (McNemar test *P* < 0.001); 30% of those who reported smoking tobacco before e-cigarette use initiation had quit after e-cigarette use initiation (former smokers). Daily tobacco cigarette consumption was reduced among those who continued to smoke from 8 (5–10) to 5 (4–8) cigarettes per day (Wilcoxon signed rank test *P* < 0.001). Current SLT use was reported by 17.9% of participants, higher than before e-cigarette use initiation (McNemar test *P* < 0.001). However, 71.0% of current SLT users reported also smoking tobacco, compared to 98.5% before e-cigarette use initiation (McNemar test *P* < 0.001).

**Table 2:** Current tobacco use patterns of study participants

	*n* (%) or median (IQR)
Current smoking tobacco	
Yes	1751 (58.4)
No	1249 (41.6)
Current daily smoking consumption	5 (4–8)
Former smokers^1^	706 (30.0)
Current SLT use	538 (17.9)
Current SLT consumption (numbers per day)	4 (2–5)
Current SLT users who also smoke tobacco	382 (71.0)

^1^Former smokers were defined as those who were smoking tobacco before e-cigarette use initiation but were not currently smoking. Proportion of those who reported smoking tobacco before e-cigarette use initiation is presented

### Patterns of e-cigarette use

The e-cigarette use patterns of the study population are presented in Table 3. A major source of information about e-cigarettes was family/friends and the internet. On average, the study population was using e-cigarettes for 20 months, with almost half reporting daily use. The majority initiated e-cigarette use with first generation devices and prefilled cartomizers. While these types of products remained popular at the time of the survey, a substantial proportion was also using third generation devices and tank-system atomizers. Refillable bottles and prefilled atomizers were the most popular container type for liquid products used. Tobacco and mint flavors were the most prevalent choices at e-cigarette use initiation; while still popular, a substantial proportion was using fruit, sweet, and nut flavors at the time of the survey. Almost 60% of participants were using at least one non-tobacco/menthol flavor, and most were using at least 2 different types of flavors regularly. E-cigarettes were purchased mostly online and from street shops.

**Table 3:** E-cigarette use patterns of study participants

	*n* (%) or median (IQR)
Where did you first hear about e-cigarettes	
Internet	1836 (61.2)
E-cigarette users’ forums	720 (24.0)
Family/friends	2086 (69.5)
Shops selling e-cigarettes (physical or online)	1044 (34.8)
TV/radio/newspapers	151 (5.0)
Healthcare professionals	46 (1.5)
Do not remember	76 (2.5)
E-cigarette duration of use	20 (12–29)
E-cigarette frequency of use	
Daily	1415 (47.2)
Weekly	1251 (41.7)
Monthly	245 (8.2)
Less than monthly	89 (3.0)
E-cigarette device used now	
1st generation (cigarette-like)	1650 (55.0)
2nd generation (eGo style)	1175 (39.2)
3rd generation (mechanical mods or variable voltage/wattage devices)	1204 (40.1)
Do not know	95 (3.2)
E-cigarette atomizers used now	
Prefilled cartomizers	1260 (42.0)
Refillable cartomizers	501 (16.7)
Tank systems with ready to use atomizer heads	812 (27.1)
Tank systems or drippers with rebuildable coils and wicks	1373 (45.8)
Do not know	96 (3.2)
E-cigarette device first bought	
1st generation (cigarette-like)	1630 (54.3)
2nd generation (eGo style)	932 (31.1)
3rd generation (mechanical mods or variable voltage/wattage devices)	343 (11.4)
Do not know	95 (3.2)
E-cigarette atomizer first bought	
Prefilled cartomizers	1279 (42.6)
Refillable cartomizers	332 (11.1)
Tank systems with ready to use atomizer heads	217 (7.2)
Tank systems or drippers with rebuildable coils and wicks	1056 (4.0)
Do not know	116 (3.9)
In which form do you buy liquids	
Prefilled cartridges	1424 (47.5)
Bottles with ready-to-use liquids	1685 (56.2)
Liquid base and flavorings (do-it-yourself)	692 (23.1)
Daily liquid consumption	5 (3–6)
Nicotine concentration in liquids	3 (3–6)
Use of non-nicotine liquids	602 (20.1)
Flavors used now	
Tobacco	1887 (62.9)
Mint/menthol	1797 (59.9)
Sweet	618 (20.6)
Nuts	674 (22.5)
Fruits	1152 (38.4)
Drinks and beverages	369 (13.2)
Other	9 (0.3)
Do not remember	8 (0.3)
Use of at least one non-tobacco/mint flavor	1780 (59.3)
Flavors used at e-cigarette use initiation	
Tobacco	1674 (55.8)
Mint/menthol	1272 (42.4)
Sweet	344 (11.5)
Nuts	345 (11.5)
Fruits	776 (25.9)
Drinks and beverages	202 (6.7)
Other	0 (0.0)
Do not remember	10 (0.3)
Number of different flavors used regularly now	
1	1034 (34.5)
2	1511 (50.4)
3	337 (11.2)
4	94 (3.1)
5	13 (0.4)
> 5	11 (0.4)
Source of e-cigarette purchase	
Online	1712 (57.1)
Department stores	1061 (35.4)
Pharmacies	243 (8.1)
Street shops	1580 (52.7)
Abroad	846 (28.2)
Other	33 (1.1)

### Self-reported effect of e-cigarettes on tobacco use status

Table 4 displays participant’s self-reported effects of e-cigarette use on tobacco use status. Smoking cessation was reported by almost a third of smoking participants, while smoking reduction was reported by an additional 41.3%. Some reported initially quitting but subsequently relapsing back to smoking, while a small minority reported increased smoking consumption after e-cigarette use initiation. Similar findings were observed among SLT users. More than one third of SLT users reported quitting, while an additional 30% reported a reduction in consumption. Again, a small minority reported an increase in SLT consumption after e-cigarette use initiation.

**Table 4:** Self-reported effects of e-cigarettes on tobacco use status of study participants

	*n* (%)
Smoking status (*n* = 2350)	
Quit smoking	706 (30.0)
Reduce smoking consumption	970 (41.3)
Quit but then relapsed	314 (13.4)
No reduction in smoking consumption	311 (13.2)
Increase in smoking consumption	49 (2.1)
SLT use status (*n* = 680)	
Quit SLT use	264 (38.8)
Reduce SLT consumption	204 (30.0)
Quit but then relapsed	149 (21.9)
No reduction in SLT consumption	61 (9.0)
Increase in SLT consumption	2 (0.3)

### Health effects of use and harm perceptions

Approximately, one third of participants reported at least one adverse symptom that they attributed to e-cigarette use (Table 5). The commonest side effect was cough, followed by headache and dry mouth/throat. More than 90% reported complete or partial resolution of the symptoms over time. More than half of participants reported improvements in their general health, breathing, olfactory and gustatory senses, and breathing after initiation of e-cigarette use (Table 6).

**Table 5:** Side effects reported by study participants after e-cigarette use initiation

	*n* (%)
**Side effects**	**Total**
Sore or dry mouth and throat	185 (6.2)
Headache	261 (8.7)
Gingivitis/gum bleeding	31 (1.0)
Dental problems	48 (1.6)
Mouth or tongue sores/inflammation	43 (1.4)
Black tongue	51 (1.7)
Nose bleeding	35 (1.2)
Cough	540 (18.0)
Bowel problems (diarrhea/constipation/pain)	5 (0.2)
Muscle cramps	22 (0.7)
Dizziness	88 (2.9)
Sleepiness	73 (2.4)
Sleeplessness	24 (0.8)
Heart palpitations	11 (0.4)
Breathing difficulties	57 (1.9)
Allergic reactions	23 (0.8)
Chest pain	119 (4.0)
Loss of appetite	15 (0.5)
No side effects	2004 (66.8)
Did the above-mentioned symptoms resolve over time?^1^	
Completely resolved	426 (42.8)
Partially resolved	501 (50.3)
Completely unresolved	69 (6.9)

^1^Proportion of those reporting any side effect

**Table 6:** Changes in health status reported by study participants after e-cigarette use initiation

	*n* (%)
After initiating EC use, have you experienced any changes in:	
Health status in general	
Worse	181 (6.0)
No change	1058 (35.3)
Better	1761 (58.7)
Smell	
Worse	177 (5.9)
No change	978 (32.6)
Better	1845 (61.5)
Taste	
Worse	111(3.7)
No change	1095 (36.5)
Better	1794 (59.8)
Breathing	
Worse	144 (4.8)
No change	1346 (44.9)
Better	1510 (50.3)
Appetite	
Worse	173 (5.8)
No change	1747 (58.2)
Better	1080 (36.0)
Sexual performance	
Worse	230 (7.7)
No change	1852 (61.7)
Better	918 (30.6)
Mood	
Worse	208 (6.9)
No change	1529 (51.0)
Better	1263 (42.1)
Memory	
Worse	193 (6.4)
No change	1574 (52.5)
Better	1233 (41.1)
Quality of sleep	
Worse	183 (6.1)
No change	1398 (46.6)
Better	1419 (47.3)
Endurance	
Worse	224 (7.5)
No change	1325 (44.2)
Better	1451 (48.4)

**Fig. 2:**
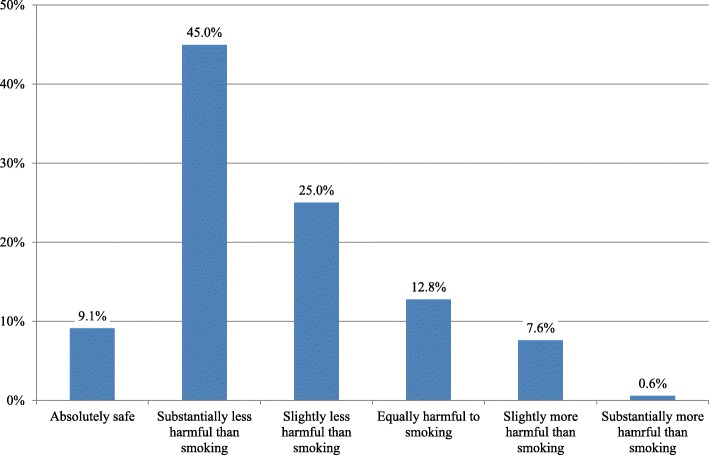
Perception of harm about e-cigarettes

Participants’ perception of harm about e-cigarettes is presented in Fig. 2. A vast majority considered e-cigarettes substantially or slightly less harmful than smoking. Less than 1 out of 10 participants considered e-cigarettes absolutely harmless while more than 20% considered them equally or more harmful than smoking.

### Correlates of having quit smoking

The results of the logistic regression analysis are presented in Table 7. Males had lower odds of having quit smoking compared to females, while higher FTCD was negatively associated with being a former smoker. E-cigarette use frequency, liquid consumption, higher nicotine concentration and perceived lower harmfulness of e-cigarettes compared to tobacco cigarettes were positively associated with being a former smoker.

**Table 7:** Regression analysis to identify correlates of having quit smoking

	Odds of being a former smoker	
	OR (95% CI)	*P* value
Age	1.00 (0.98–1.01)	0.573
Gender		
Female (point of reference)		
Male	0.58 (0.43–0.80)	0.001
Education		
Less than Senior School Certificate (point of reference)		
Secondary/High School Certificate	1.95 (0.21–18.31)	0.560
Graduate/postgraduate	3.07 (0.33–28.20)	0.322
Professional degree	4.38 (0.48–40.31)	0.192
Smoking duration	1.00 (1.00–1.00)	0.399
Smoking before e-cigarette use initiation	1.03 (0.99–1.07)	0.156
FTCD	0.86 (0.81–0.92)	< 0.001
E-cigarette use duration	0.99 (0.98–1.00)	0.017
E-cigarette use frequency		
Less than monthly (point of reference)		
Daily	2.67 (1.28–5.58)	0.009
Weekly	3.04 (1.45–6.38)	0.003
Monthly	1.70 (0.76–3.81)	0.199
E-cigarette liquid consumption	1.33 (1.27–1.39)	< 0.001
Nicotine concentration	1.06 (1.02–1.10)	0.007
Number of flavors used		
1 (point of reference)		
2 or more	1.25 (0.99–1.57)	0.062
Flavors type used		
Tobacco/mint/menthol only (point of reference)		
At least one non-tobacco/mint/menthol	0.95 (0.76–1.19)	0.669
Perception of harm		
Equally/more harmful than smoking (point of reference)		
Harmless/less harmful than smoking	1.80 (1.34–2.41)	< 0.001

*FTCD* Fagerstrom Test for Cigarette Dependence

## Discussion

To the best of our knowledge, this is the first study to examine the characteristics of vapers in India. Participants were mostly smokers and SLT users, in agreement with other studies of adult populations showing that e-cigarette use is predominantly observed among current or former smokers [22, 26, 27]. The main results of this survey indicate that e-cigarettes could potentially be an effective partial or complete substitute for tobacco use for some smokers and SLT users in India. While this should be considered in the context of limited smoking cessation interventions in India, the government has recently decided to ban these products.

A substantial proportion of the study population experienced benefits in terms of reduction or quitting smoking. A similar proportion reported smoking reduction and cessation in the Eurobarometer study, although that was a population-representative sample [27]. Frequent e-cigarette use was one of strongest correlates of being a former smoker. This is expected, considering that experimental or occasional use of any smoking cessation aid is unlikely to be substantially effective. Frequent e-cigarette use could indicate motivation and intention to use them as smoking cessation aids or could be the result of completely substituting for smoking. In any case, our findings are in agreement with several studies showing that frequent e-cigarette use is associated with smoking cessation [28–31]. Higher nicotine concentrations were also positively associated with being a former smoker. Nicotine use with e-cigarettes is important in order to successfully substitute for smoking [22, 32], and it is expected that tobacco users would need high enough amounts to satisfy their nicotine cravings. While nicotine use by never smokers carries a risk of dependence, it is important for smokers to use e-cigarettes with sufficient nicotine delivery. Another factor that affects nicotine delivery is the type of device used. A large proportion of vapers in this study initiated e-cigarette use with first generation devices, and many were still using them at the time of the survey. These devices are known to deliver a limited amount of nicotine compared to more advanced products [33–35]. This may be due to India being a small and developing market for these products, with lack of information to guide choice of devices compared to other countries where e-cigarettes have been available more widely and for a longer time. Finally, perceiving e-cigarettes as less harmful than smoking was associated with being a former smoker, in agreement with a previous study [36]. While it is expected that smokers need to trust any alternative-to-smoking product in order to succeed in quitting, it is unclear whether the perceptions observed herein were the reason for, or the result of, quitting smoking.

Less than 35% of participants experienced some self-reported side effects after e-cigarette use initiation. The most common were cough and dry mouth and throat. These have been observed in previous studies too [22, 37, 38] and are commonly attributed to the humectant and irritating effects of propylene glycol and glycerol. At the same time, they perceived significant benefits in physiologic functions, mainly breathing and olfactory and gustatory senses, and overall physical status were reported. Obviously, the convenience sampling design of this study is an important limitation; thus, the findings should be interpreted with caution and may not be representative of the experience of all people who try and use e-cigarettes. Still, the findings are consistent with limited clinical studies showing benefits among smokers who switch to e-cigarettes [39, 40].

India has the second lowest quit rate among GATS-2 countries surveyed at the end of 2017 [6]. Despite a large proportion of smokers reporting a desire to quit, few make a quit attempt each year, and a small minority achieve abstinence [1, 41]. GATS-2 data for India show that only 50–55% of tobacco users intended, and about 39% attempted, to quit [42]. Obviously, a much smaller proportion is successful in quitting. Lung cancer, the most prevalent type of cancer today [43], is highly prevalent in India. It is the most prevalent cancer among males, and the seventh most prevalent among females [44], which correlates well with the differential tobacco smoking prevalence among males and females [6]. The most recent demographic data indicate that the recorded decline in tobacco usage in India noted in the intervening 7 years between GATS-1 and GATS-2 is probably insufficient to strongly reduce the prevalence of disease associated with tobacco use. Both smoking and SLT use continue to be a serious public health concern in India. Currently, available tobacco cessation services are inadequate to care for the estimated 267 million tobacco users, including over 100 million smokers [6, 11]. This lack of capacity is compounded by a lack of capability among healthcare professionals, who are mostly inadequately trained, to deliver tobacco cessation advice. The use of tobacco by healthcare professionals is also prevalent, potentially demotivating provision of effective cessation advice to smokers [10]. The existing tobacco control program needs to be strengthened by including additional tobacco cessation aids. The present study suggests that there is a potential for e-cigarettes to substitute for smoking and SLT use among Indian tobacco users. Clinical trial evidence is emerging supporting the smoking cessation efficacy of e-cigarettes [17]. On the other hand, the Indian government has recently banned e-cigarette sale due to concerns that they are not risk-free and that their availability could trigger use by never smokers and youth, among others. To benefit public health, a balance is needed between encouraging smokers who are unable or unwilling to quit with approved methods to use e-cigarettes as smoking cessation aids and preventing its use by non-smokers. Regulations that achieve this balance do exist, for example in Europe, where e-cigarettes are regulated through the Tobacco Products Directive as consumer products with specific restrictions [45]. It is unlikely that the implemented ban in India represents a balanced approach, especially when considering that there has been no research on the profile of Indian vapers, their tobacco use profile before and after e-cigarette use initiation, and how the ban will affect their future tobacco use. While one of the main reasons behind the ban is to prevent youth use, to the best of our knowledge and belief, no study has examined use of e-cigarettes among Indian youth. Youth use in other countries has been largely experimental and confined to youth who were already smoking or had tried tobacco cigarettes in the past [46, 47]. Other issues, such as the development of a black market with uncontrolled product quality, have not been considered either. This study provides preliminary insight into the use of e-cigarettes by Indian vapers, but research should be expanded by examining population-representative samples and assessing the impact of the ban at a population level.

A limitation of this study is the convenience sampling design, which cannot ensure that the profile of the vapers examined represent the average Indian consumer. Still, this remains the first study examining patterns of e-cigarette use in India. Additionally, the tobacco use status of participants was self-reported and not objectively verified, although this is common for large surveys. Data on tobacco use patterns and dependence before e-cigarette use initiation were accessed retrospectively, and there is a possibility for recall bias. Finally, despite participants responding on whether e-cigarettes helped them quit or reduce tobacco use, this is a cross-sectional study, and causal relationships cannot be confirmed. Future prospective or longitudinal studies are needed to confirm the suggested result.

## Conclusion

In conclusion, and considering the above-mentioned limitations, Indian e-cigarette users who participated in the study were predominantly smokers and SLT users, with a significant proportion of them managing to quit or reduce their tobacco consumption. Minimal side effects were experienced, while some health benefits were also reported. Our findings highlight the potential of e-cigarettes to be an additional option for tobacco control. This may be especially relevant for countries such as India, which have (a) high tobacco-related health burden, (b) complex tobacco landscape, and (c) inadequate infrastructure and resources for offering tobacco cessation to help smokers and SLT users quit. While the opportunity for India seems to have been missed due to the implemented ban, public health authorities should encourage additional research and consider suitable modifications in the regulatory framework if findings support such a need.

## Data Availability

Most data are contained in the manuscript itself. Additional information, if required, shall be made available.

## Abbreviations

SLT: Smokeless tobacco
E-cigarette: Electronic cigarette
WHO: World Health Organization
GATS: Global Adult Tobacco Survey
IQR: Interquartile range
